# The Importance of Controlling Terminal Complement Activity and Intravascular Hemolysis in Paroxysmal Nocturnal Hemoglobinuria (PNH)

**DOI:** 10.1002/ajh.27556

**Published:** 2024-12-09

**Authors:** Anita Hill, Christophe Hotermans, Gianluca Pirozzi

**Affiliations:** ^1^ Alexion, AstraZeneca Rare Disease Boston Massachusetts USA

To the Editor,

Patient safety is paramount and ingrained in our mission at Alexion, and with our leadership in complement biology for over 30 years, we strive to provide the most efficacious and safe therapeutic products for patients. It must be remembered that the most significant risks to patients with paroxysmal nocturnal hemoglobinuria (PNH) come from intravascular hemolysis (IVH) and thrombosis, and these are mediated by terminal complement activity.

Risitano et al. details the experience of three patients previously treated with vemircopan monotherapy, who were switched to ravulizumab when the clinical trial NCT04170023 was discontinued [1]. The authors conclude that hemolysis following discontinuation of effective proximal complement inhibitors poses a significant clinical risk that cannot be fully mitigated by switching to C5 inhibitors and recommend pursuing an alternative proximal complement inhibitor. The phenomenon described is expected, having first been seen in the PEGASUS trial where patients were managed supportively with transfusions when necessary [2]. The current correspondence contributes further to the evidence of this phenomenon for patients when they are discontinued from proximal complement inhibition.

Terminal complement inhibitors have transformed the natural history of patients suffering from PNH by providing effective disease control over the last two decades with a well‐established safety profile. Furthermore, long term efficacy and safety data have demonstrated reduced risk of thrombosis, improved survival rates, and control of IVH with low and less severe rates of breakthrough intravascular hemolysis (BT‐IVH) in patients with PNH. The improvement in survival for patients with PNH has been achieved by terminal complement inhibition alone, reinforcing the critical role that terminal complement plays in the progression of this disease.

In the phase II, proof of concept study evaluating safety and dosing of vemircopan (a proximal inhibitor) as a monotherapy in patients with PNH (NCT04170023), the trial was terminated due to inadequate and inconsistent IVH control, which results in poor disease outcomes. Lactate dehydrogenase (LDH), a marker of terminal complement activity, was not consistently and sufficiently suppressed amongst all patients resulting in LDH excursions (defined as LDH values > 2× upper limit of normal [ULN]) and unacceptably high rates of BT‐IVH. This raised concerns about potential risk for thrombotic events and premature mortality in this group of patients. Control of terminal complement activity and IVH, as reflected by LDH, is the primary aim for patients with PNH to prevent significant morbidity and mortality. In the best interest of patients' safety, the clinical trial was discontinued with due consideration given to availability of terminal complement inhibitors. A manuscript that reports the phase 2 clinical trial results with vemircopan (NCT04170023) is in development.

Risitano et al. describe patients A and C as having “massive, acute, extravascular hemolysis [EVH]” and patient B as having “low‐grade residual IVH.” It is critical to distinguish between the two types of hemolysis in patients with PNH. As confirmed by the literature, LDH > 1.5× ULN is a predictor of thromboembolisms (TE), and TE is a predictor of mortality [3]. The terminal complement pathway forms the membrane attack complex (MAC) through non‐enzymatic processes [4]. This in turn generates one MAC formed for each C5 molecule that escapes inhibition, and results in limited BT‐IVH. In contrast, proximal inhibitor pathways employ an enzymatic activation cascade with the potential for enormous amplification potential. With incomplete inhibition with proximal inhibitor monotherapies, there is potential for the formation of C5 convertases that cleave several C5 molecules and generate many MACs, leading to potentially massive BT‐IVH. This is also reflected by Risitano et al. as LDH did not rise above 2× ULN upon switching to ravulizumab therapy, clinically demonstrating the proposed pathophysiological process. While the patients described are in a unique situation than those with untreated PNH (having received treatment with a proximal inhibitor), controlling LDH remains paramount. These findings further support patient safety and protection with ravulizumab from the serious consequences of PNH resulting from terminal complement activation. Ultimately, a high LDH reflects high disease activity and risk of thrombosis, renal impairment, pulmonary hypertension, and death. An LDH > 1.5× ULN will detect 96% of patients suffering a thrombosis [5]. Therefore, the primary goal of PNH treatment is to achieve LDH ≤ 1.5× ULN.

A focus primarily on hemoglobin does not account for the role that terminal complement activity and IVH play in the development of the serious complications in PNH. Clinical trial experience and real‐world cases are beginning to emerge, underscoring the seriousness of BT‐IVH with proximal inhibitor monotherapy and will continue to demonstrate that if the role of terminal complement in the pathophysiology and progression of PNH is forgotten, patient safety is at risk. Once the primary goal of complete and sustained terminal complement inhibition has been achieved for the safety and survival of patients, and if patients experience symptomatic anemia, this then needs to be investigated and managed appropriately. We agree that improving quality of life is important, but not at the cost of compromising control of terminal complement activity and IVH, to reduce the risk of organ damage and thrombosis, and thereby improving survival.

For the subset of patients who continue to experience symptoms associated with EVH, dual complement inhibition may be considered as an important therapeutic option. Proximal complement inhibition helps address the effects of symptomatic hemolytic anemia, while the terminal complement inhibitor maintains the essential control of terminal complement activity and IVH—the primary goal of treatment that should not be compromised.

## Ethics Statement

The authors have nothing to report.

## Consent

The authors have nothing to report.

## Conflicts of Interest

AH, CH, GP are employees of Alexion, AstraZeneca Rare Disease and own stock in AstraZeneca.

## Data Availability

The authors have nothing to report.
